# Insulin-like Peptide Receptor (ILPR) in the Cuttlefish *Sepiella japonica*: Characterization, Expression, and Regulation of Reproduction

**DOI:** 10.3390/ijms232112903

**Published:** 2022-10-26

**Authors:** Zhenming Lü, Yantao Liu, Jun Yan, Yao Zhang, Li Gong, Bingjian Liu, Jing Liu, Zhijin Xu, Liqin Liu

**Affiliations:** 1 National Engineering Research Center for Marine Germplasm Resources Exploration and Utilization, Marine Science and Technology College, Zhejiang Ocean University, Zhoushan 316000, China; 2 Zhejiang Marine Fisheries Research Institute, Zhoushan 316000, China; 3 Zhoushan Fisheries Research Institute of Zhejiang Province, Zhoushan 316000, China

**Keywords:** cephalopod, reproduction, ILPR, RNA interference, transcriptome sequencing

## Abstract

Insulin-like peptide receptor (ILPR) can effectively regulate ovarian development in invertebrates, but its effect in cuttlefish has not been reported. We isolated and characterized a ILPR gene from *Sepiella japonica*, referred to as *SjILPR*. This gene displayed significant homologies to Octopus bimaculoides ILPR, and contained all typical features of insulin receptors and tyrosine kinase domain structure. *Sj*ILPR is expressed in all detected tissues, with the highest expression in the ovary. During ovarian development stages, its expression levels in the ovary, pancreas, and liver were correlated to the female reproductive cycle. After the silencing of *Sj*ILPR in vivo, comparative transcriptome analysis identified 4314 differentially expressed genes (DEGs) in the injected group, including 2586 down-regulated genes and 1728 up-regulated genes. Kyoto Encyclopedia of Genes and Genomes (KEGG) pathway analyses revealed that 832 DEGs were assigned to 222 pathways, many pathways of which were related to gonadal development. Four down-regulated genes relevant to ovarian development (Vitellogenin 1, Vitellogenin 2, Cathepsin L1-like, and Follistatin) were selected to confirm the accuracy of RNA-seq data by qRT-PCR. These results showed that *SjILPR* might regulate ovarian development to control reproduction by affecting the expression of the relevant genes in female *S. japonica*.

## 1. Introduction

The cuttlefish *Sepiella japonica*, an important commercial cephalopod, distributed in the East China Sea, has been regarded as one of the four major marine fishery species of China since the 1970s. However, over-fishing has led to the decline in the wild *S. japonica* population. With the development of artificial breeding techniques, aquaculture techniques have been successfully utilized for this species in China. Moreover, *S. japonica* exhibits sexual precocity under an artificial environment [1]. A key reason resulting in sexual precocity is the premature ovary development of females. Thus, it appears essential to understand the regulatory mechanisms of ovarian development in controlled conditions. In mollusk species, ovarian development is a complex process that is regulated by multiple factors [2,3,4], among which, insulin-like peptide (ILP) and insulin pathways play key roles in the process [5].

Insulin-like peptide (ILP), a member of the insulin-like superfamily, has been proven to regulate reproduction through a conserved insulin signing pathway in invertebrates [6,7,8]. For example, in *Schistocerca gregaria*, *Scg-*IRP (insulin-related peptide) transcript levels elevated during vitellogenesis and growth of the oocytes [9]. In addition, knock-down of the *Scg-*IRR (insulin-related peptide) declined Vitellogenin transcript levels and oocyte growth [8], which suggest ILP regulates reproduction via involvement in the control of vitellogenesis. In the mosquito *Aedes aegypti*, ILPs positively regulate vitellogenesis [7], combined with the finding that mosquito ovaries express IR (insulin receptor) and PKB homologs, these results suggest that ILP regulated ovary maturation via activating IR through the PI3K/AKT pathway [6,7,10].

In mollusks, since the first identification of molluscan insulin-related peptide (MIP) in *Lymnaea stagnalis* [11], multiple ILPs have been reported in different species [12,13,14]. However, in most mollusks studied, only one insulin-like peptide receptor (ILPR) has been identified despite multiple ILPs [15]. Additionally, this receptor shares great structural and functional similarities to IR of vertebrates [16,17,18,19,20]. Studies in the pond snail *L. stagnalis* further proved that four different ILP may play their functional roles by binding to the same molluscan ILPR [16]. The IRR of *Pinctada fucata* is widely expressed in multiple tissues and developmental stages, which indicates its involvement in regulating the development of embryos in *P. fucata* [19]. In the Pacific oyster *Crassostrea gigas*, *Cg*-ILP, and *Cg*-IRPs are mainly expressed in the gonadal area [14,21]. Furthermore, in *C. gigas*, gonadal area also expressed three potential elements of the insulin pathway (Cg-Ras, Cg-Pten, and Cg-P70S6K), these results suggest ILP regulates reproduction by activating the insulin-signaling pathway in a mollusk species [22].

In *S. japonica*, no ILPR has been identified, but the ILP has been reported by our lab (GenBank: MK611805) [23]. Here, to evaluate the physiological role of ILPR in reproduction regulation. Firstly, we cloned and characterized the complete cDNA sequence encoding ILPR from *S. japonica*. Next, we detected the subcellular localization and examined its spatio-temporal expression profiles by quantitative real-time PCR (qPCR). Finally, we performed a comparative transcriptome analysis of the ovary after the silencing of *Sj*ILPR to identify the differentially expressed genes and essential pathways related to gonadal development. Four down-regulated genes were selected to verify the accuracy of RNA-seq data by qRT-PCR. Our results will help to understand the regulatory mechanisms associated with ovarian development and the reproductive process, which serve to guide the artificial breeding of cuttlefish.

## 2. Results

### 2.1. Cloning and Sequence Analysis of SjILPR

**Figure 1:**
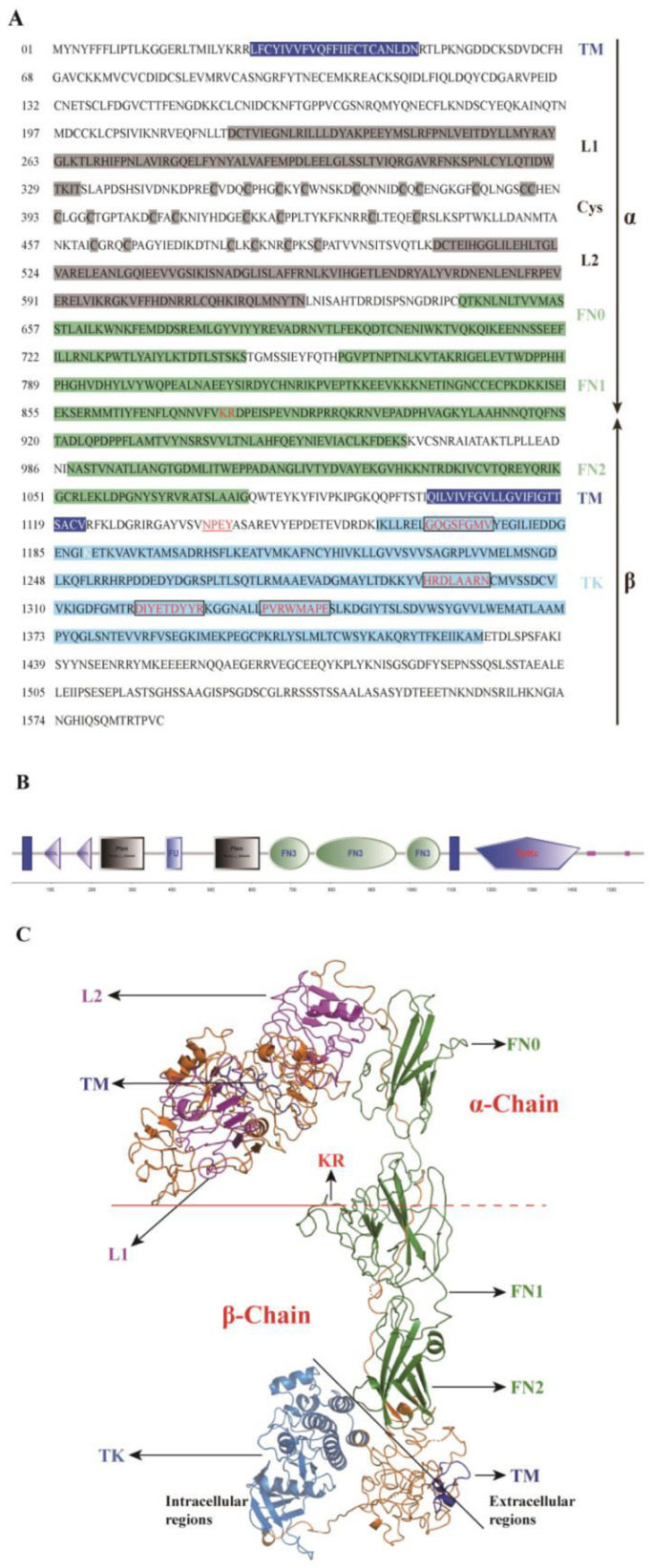
Amino acid sequence (**A**), structural feature (**B**), and three-dimensional (3D) structure (**C**) of *SjILPR*. (**A**) The dibasic site KR is highlighted in red script. The transmembrane domains are shown in white script on blue background. Indicated are the ‘L1–Cys–L2′ structure (gray background), three fibronectin III domains (light green background), and the tyrosine kinase domain (light blue background). Juxtamembrane NPEY motif is highlighted in the red script and underlined. Catalytically important residues of the TK domain are highlighted in red script and black rectangles. (**B**) Smart domain architecture of *SjILPR*: the blue rectangle depicts the transmembrane domain; Recep_L_domain indicates the receptor L domains; FU, furin-like repeats; FN3, fibronectin type 3 domain; and TyrKc, the tyrosine kinase catalytic domain. (**C**). The predicted 3D structure of *SjILPR*, is structurally based on *Homo sapiens* insulin receptor.

The completed ILPR cDNA sequence isolated from *S. japonica* was 5288 bp long, corresponding to an open reading frame (ORF) of 4764 bp, a 487 bp 5′-untranslated region (UTR), and a 37 bp 3′-UTR. After translating, the ORF encoded a 1587 amino acid sequence. The deduced protein sequence showed 67.3% amino acid sequence identity to ILPR from *Octopus bimaculoides* (Supplementary Table S3). Examination of this sequence identified many of the typical characteristics of ILPRs, including two transmembrane region (TM) domains (aa 27–49 and 1100–1122), the receptor L domains (aa 220–332 and 507–622), separated by one furin-like domain (aa 385–427), three fibronectin type 3 domains (aa 644–747, 760–965, 988–1075), a tyrosine catalytic domain (aa1160–1427) and a potential dibasic cleavage site (KR, aa 876–877) (Figure 1A,B). In further structural analyses of the intracellular domain, we found several major characteristic domains that are crucial for protein kinase activity and downstream signalings, such as an NPXY (where X is any amino acid) motif (N_1138_PDY_1141_), a GXGXXG-motif (G_1167_QGSFGMV_1174_), two consensus sequences (D_1295_LAARN_1300_ and P_1204_VRWMAPE_1211_), and a signature pattern for class II RTKs (D_1320_IYETDYYR_1328_) (Figure 1A). The C-terminal domain of the *Sj*ILPR was longer, and showed low complexity. Based on these key features, the sequence obtained from *S. japonica* was named *Sj*ILPR (GenBank accession number: MK611806).

The putative 3D structure of the ILPR protein was obtained at the I-TASSER-MTD sever using an automatically matched template (Homo sapiens insulin receptor, PDB 7LHW) and showed a similar structure (TM score, 0.57) (Figure 1C). This similarity also proved the conserved structure of *Sj*ILPR at the tertiary level.

### 2.2. Phylogenetic Analysis of SjILPR

**Figure 2:**
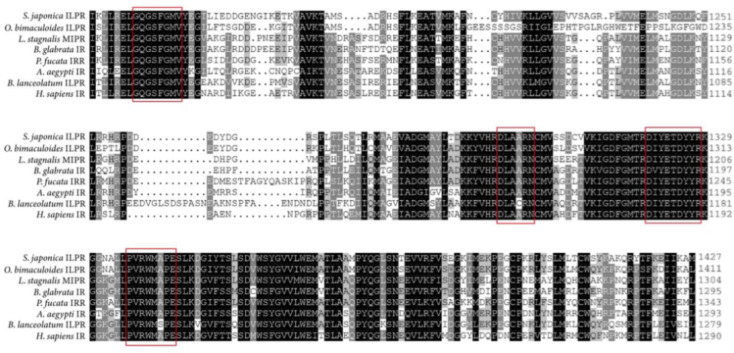
Comparison of the tyrosine kinase catalytic domain of *SjILPR* with TK domains of IRs from different species. Conserved residues are between the *SjILPR* and the other seven IRs are shaded in black, grey shading indicates identity with at least one of the seven sequences. Catalytically important residues of the TK domain are red boxed. The accession numbers are shown in Supplementary Table S2.

**Figure 3:**
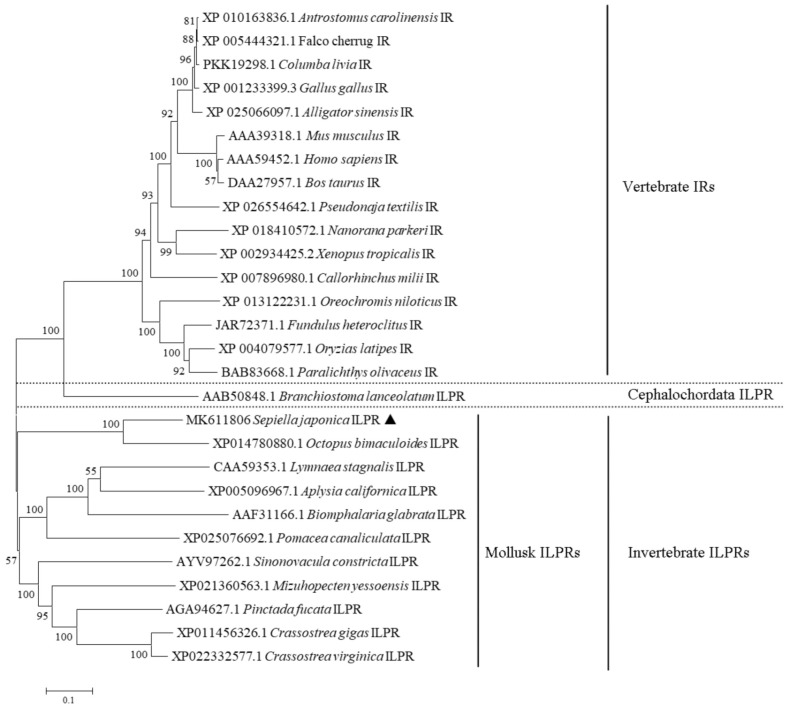
Phylogenetic tree of IR amino acid sequences of vertebrates and invertebrates. The tree was constructed by MEGA7.0 using the neighbor-joining method with 1000 bootstrap replicates. The triangle indicates *Sj*ILPR.

Multiple sequence alignment analysis showed that *S. japonica* ILPR displayed 36.9 to 66.3% amino acid identity with other vertebrate and invertebrate species (Supplementary Table S3). As the most conserved region corresponding to the TK domain between species, several essential motifs such as the invariant K1196, the consensus sequences GQGSFGM, HRDLAARN, PVRWMAPE, and DIYETDYYR exhibited high homology (Figure 2). The phylogenetic tree showed that *Sj*ILPR was clustered into one clade with the ILPs of other mollusks, while the IRs of cephalochordate and vertebrate formed other clade (Figure 3). In addition, *Sj*ILPR was closely related to ILPR of *Octopus bimaculoides*, which also belongs to Cephalopoda and shows the highest identity to *Sj*ILPR.

### 2.3. Subcellular Localization of SjILPR

**Figure 4:**
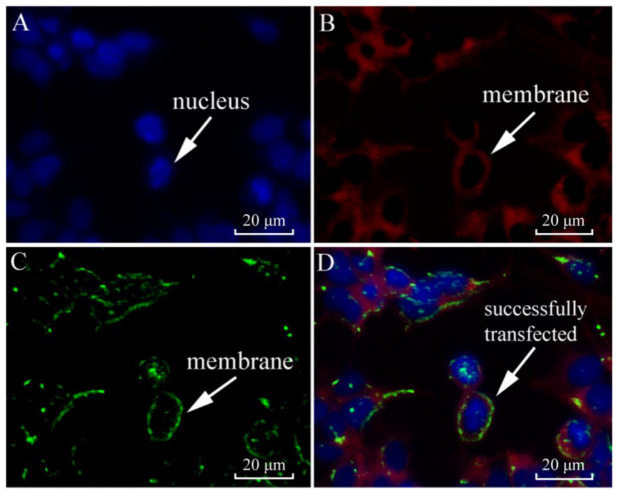
Subcellular localization of *SjILPR*. (**A**) Nucleus of HEK293T cells were stained with DAPI (shown in blue). (**B**) Plasma membranes of cells were stained with DiI (shown in red). (**C**) *SjILPR*-EGFP fusion protein was shown in green. (**D**) Localization of *SjILPR* was depicted in yellow. The nucleus and plasma membrane are indicated with white arrows in (**A**–**D**).

The subcellular localization of *Sj*ILPR was examined in transfected HEK293T cells. As shown in Figure 4D, *Sj*ILPR was localized in the plasma membrane (as indicated by the white arrows). This result indicated the characteristic of the transmembrane receptor.

### 2.4. Expression of SjILPR in Different Tissue

**Figure 5:**
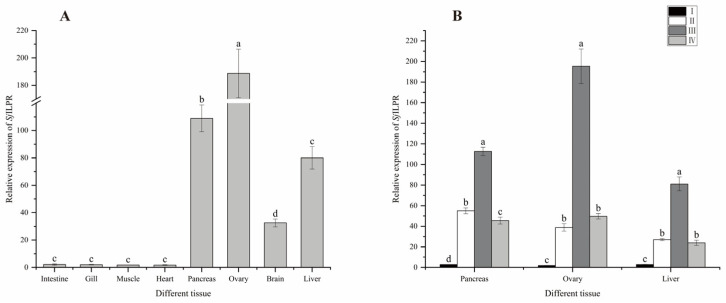
Expression of *SjILPR* in different tissues and ovarian development stages. Relative expression of *SjILPR* is quantified and compared between different tissues (**A**). Expression levels of *SjILPR* in the ovary, pancreas, and liver tissues during ovarian development stages are quantified. Comparison of *SjILPR* expression levels between different stages in these three tissues (**B**). The results are expressed as mean ± SD (n = 7). Mean with different letters indicates significance (*p* < 0.05).

The expression of *SjILPR* was examined in different tissues by qRT-PCR. As shown in Figure 5A, it was expressed in all investigated tissues, with the highest expression in the ovary, and moderate expression level in the pancreas, liver, and brain. However, in the other four tissues (heart, muscle, gill, and intestine), it was weakly expressed without significant difference (*p* < 0.05).

### 2.5. Expression of SjILPR during the Different Ovarian Development Stages of Female S. japonica

As shown in Figure 5B, the *Sj*ILPR expression profiles in the three most abundant tissues (ovary, pancreas, and liver) during ovarian development stages were similar. The mRNA transcript of *Sj*ILPR increased from stage I to stage III reaching the peak value at stage III, then decreased, and kept at a low level at stage IV (*p* < 0.05).

### 2.6. Transcriptome Analysis of the Ovary after the Silencing of SjILPR

**Figure 6:**
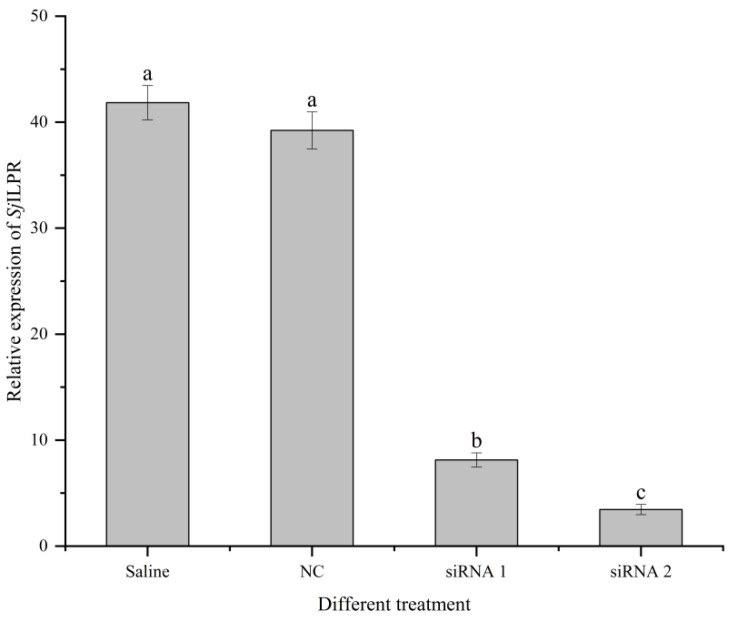
The expression of *Sj*ILPR after the silencing *Sj*ILPR. Relative expression of *Sj*ILPR was analyzed and compared between control, Saline, siRNA1, and siRNA2 groups. The results are expressed as mean ± SD (n = 8). Mean with different letters indicates significance (*p* < 0.05).

To detect the effect of RNAi, we examined the expression level of *Sj*ILPR after *SjILPR* knockdown. As shown in Figure 6, *Sj*ILPR expression in the ovary was effectively reduced by RNAi targeting of *Sj*ILPR when compared to control, with siRNA2 having the better inhibitory effect (*p* < 0.05). Thus, we compared the transcriptional profiles between the siRNA2 injected and control groups.

Among the 103,117 unigenes obtained, a total of 37,171 (36.04%) unigenes were subjected to annotation by matching sequences against NR, NT, KO, Swiss-Prot, PFAM, GO, KOG/COG databases using BLASTX search. As shown in Supplementary Table S4, 25,602, 8201, 12,276, 17,718, 28,190, 28,190 and 6637 unigenes matched in the NR, NT, KO, Swiss-Prot, PFAM, GO, KOG database, respectively.

After sequencing by the Illumina platform, the reads were assembled using the Trinity software. A total of 20.63 Gb clean bases (10.09 Gb clean reads in the control group, and 10.54 Gb in the treatment group) was obtained from the raw data. The Q20 of the clean reads in the two cDNA libraries were 96.85% for the control group, and 96.77% for the treatment group (Table 1). We generated a total of 103,117 unigenes with an average length of 1007 bp (Figure S1).

**Figure 7:**
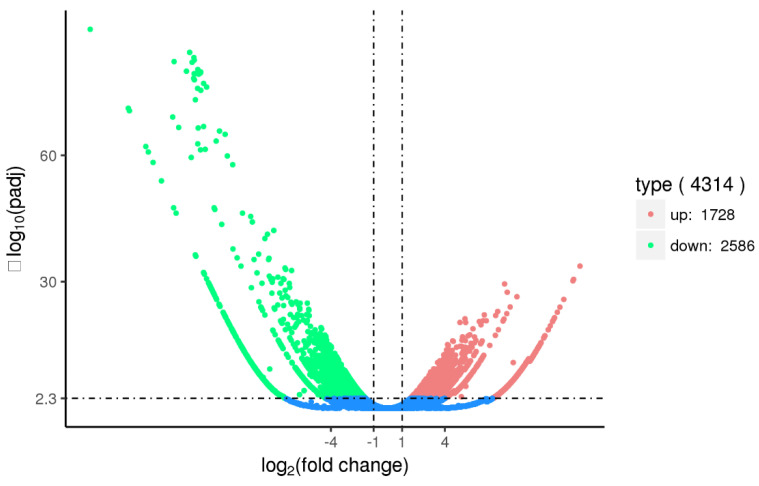
Gene transcription profile volcano plot of the control and treatment group. Red points indicate up-regulated genes. Green points indicate down-regulated genes.

**Figure 8:**
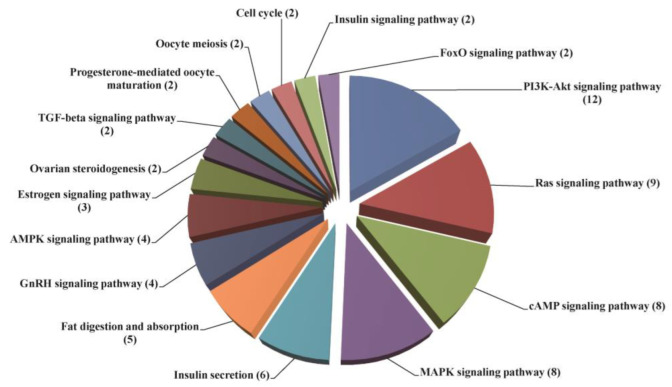
Pathway assignments of DEGs involved in gonadal development based on the information extracted from the KEGG database. The number in brackets represents the number of the unigenes mapped to each pathway.

**Figure 9:**
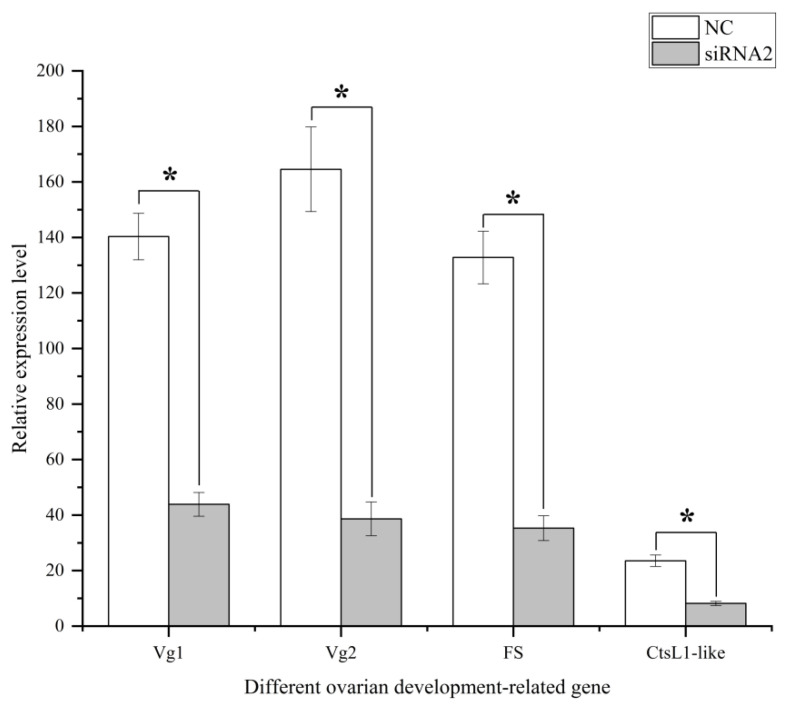
Expression analysis of ovarian development-related genes in the ovary after the silencing of *Sj*ILPR. Expression levels of each gene are compared to their respective control levels. The results are expressed as mean ± SD (n = 8). Statistical significance is indicated by asterisks (*).

At 48 h after the silencing of *Sj*ILPR, we identified 4314 differentially expressed genes (DEGs) between the control and injected groups, of which, 1728 were up-regulated, and 2586 were down-regulated (Figure 7). Among these 4314 DEGs, 832 DEGs were assigned to 222 pathways. Among these 222 pathways, some pathways were associated with gonadal development, ovarian maturation, and reproductive endocrinology (Figure 8). After the silencing of *Sj*ILPR, the expression level of ovarian development-related DEGs including Vitellogenin 1 (Vg1), Vitellogenin 2 (Vg2), Cathepsin L1-like (CtsL1), and Follistatin (FS) decreased. To validate the expression of DEGs, we further examined expression levels of four down-regulated genes in samples from the control and injected groups by qRT-PCR. As shown in Figure 9, the expression levels of these four genes were significantly reduced after *Sj*ILPR knockdown (*p* < 0.05), which was identical to the results from RNA-seq data.

## 3. Discussion

The insulin-signaling pathway has been indicated in the involvement in regulating reproduction in invertebrates, while information regarding its role in the reproduction regulation of mollusks, such as cuttlefish, remains limited. In the present study, we identified an ILPR gene in *S. japonica.* Structural analysis showed that *Sj*ILPR possessed a conserved tyrosine kinase domain, and belongs to class II RTKs [24]. Alignment of the putative *Sj*ILPR amino acid sequence with other IRs from different species showed similar structural organization, containing a Cys-rich region, a potential cleavage site, a TM domain, and a TK domain (Figure 1A,B). However, there were some differences between the *Sj*ILPR and other IRs. Generally, DIR and mammalian IRs contain a tetrabasic site known to be important for the receptor where it was cleaved to mature α2β2 hetero-tetrameric structure [25]. However, *Sj*ILPR lacks such a tetrabasic cleavage motif. Instead, a dibasic site (KR) is present at a corresponding position, which is possibly the site of cleavage of the receptor into active form in *S.japonica*. Another important difference observed among IRs was the presence of the two transmembrane regions in *Sj*ILPR (Figure 1A,B). While for most IRs, there is one TM residue on the β-subunit, which is thought to be involved in anchoring the receptor in the plasma membrane, transmitting the signal into the interior of the cell, and activating the RTK domain. However, a transmembranal domain was identified in *Sj*ILPR near the N-terminal of the α-subunit, as recorded with the other IRs (e.g., *D. pulex* and *M. rosenbergii*) [26,27]. Furthermore, the function of such a domain at the N-terminal of the putative protein sequence remains unknown.

The highly conserved ‘L1–Cys–L2’ structure, composed of a Cys-rich region and two flanking regions (L1 and L2), is important for ligand binding [16]. Near the juxtamembrane region, a characteristic motif (NPFY) was essential for functional interaction between ILPR and insulin receptor substrate (IRS) factors [28]. *Sj*ILPR possessed a mostly conserved TK domain, which is the vital factor for the generation and translocation of signals [29,30]. This region contained some characteristic structures, which are essential for protein kinase activity [18]. For example, a consensus sequence (GQGSFGMV) was important for ATP binding, and the invariant V_1174_ was necessary for the correct positioning of conserved glycines and their interaction with the ATP [31]. Moreover, the TK domain also has another two consensus sequences, HRDLAARN and PVRWMAPE, which confirms the tyrosine kinase nature of the mollusk receptor [31]. Furthermore, this receptor contained the auto-phosphorylation site (YETDYYR), which is essential for the up-regulation of receptor catalytic activity [32].

Based on the subcellular localization analyses, *Sj*ILPR protein is localized in the plasma membrane of cells (Figure 4), which is consistent with the results from immunocytochemistry in *Aedes aegypti* [6]. Combined with the structural organization containing transmembrane domains (Figure 1A,B), these results confirmed that *Sj*ILPR belongs to the membrane receptor.

*Sj*ILPR is expressed in all examined tissues, which indicated a wide distribution and diverse functions in *S. japonica* (Figure 5A). It is consistent with previous studies in *Aplysia californica* [17], *C. gigas* [5], and *Pinctada fucata* [19]. The highest expression level in the ovaries strongly suggested the possible role of this receptor in regulating reproduction. The high expression level of *Sj*ILPR in the pancreas may be related to the paracrine function of *ILPR* in digestion [19]. The liver is also a major insulin-sensitive organ, and regulates metabolism [33]. So, the relatively higher expression level of *Sj*ILPR in the liver might indicate involvement in a metabolic activity besides reproduction [34]. The *Sj*ILPR was also expressed in the brain tissue (Figure 5A), which is in good agreement with the result that *Aplysia californica* IRR is expressed abundantly in the bag cell neurons. Because bag cell neurons function to initiate egg laying, it is possible that ILP may regulate the triggering of egg laying and its associated behaviors [17]. Moreover, *Sj*ILPR was also expressed in several potential target organs of insulin-like signalings, such as the intestine, heart, muscle, and gill [35,36]. This distribution might be in line with the biological roles of ILP on growth, metabolism, and development.

The expression of *SjILPR* throughout the reproductive cycle was in line with ovarian maturation, which indicated its role in regulating the reproduction of *S. japonica* (Figure 5B). This expression pattern agreed with ILP effects on ovary development, previously demonstrated in *A.aegypti* [6], oyster *C. gigas* [5]. *Sj*ILPR was expressed at a low level during stage I (previtellogenic arrest stage) (stage), which is consistent with previous studies in *C. gigas* [22] and *P. fucata* [19]. After stage I, its expression increased, peaked at stage III, then declined. In most mollusks, the gonad is a non-permanent organ undergoing a reproductive cycle [37]. Egg-yolk accumulation (vitellogenesis) in oocytes is a critical event during ovary development [38]. In female *S. japonica*, the initiation phase of vitellogenesis begins at stage II, and the yolk accumulation occurs during stage III [2]. The expression pattern of *Sj*ILPR matched the periodic ovarian development of cuttlefish. Furthermore, combined with our previous study that *Sj*ILP expression level was associated with ovary development in female *S. japonica* [23], this result suggested that *Sj*ILPR plays an important regulatory role in the ovarian development in Cephalopod species.

Compared to the control group, 4314 DEGs were identified in the injected group, including 2586 down-regulated genes and 1728 up-regulated genes. KEGG pathway analysis showed that 832 DEGs were assigned to 222 pathways, and some pathways were related to gonadal development. They include the cell cycle, oocyte meiosis, progesterone-mediated oocyte maturation, ovarian development, and GnRH signaling pathways (Figure 8). These pathways and related genes also have been proven to be involved in gonadal development in other species [39,40,41,42]. In addition, some down-regulated genes related to ovarian development (e.g., Vitellogenin1, Vitellogenin2, Cathepsin L1-like, and Follistatin) were also found in our data, which suggested *Sj*ILPR may be involved in ovarian development via affecting the expression of these genes.

Ovarian development is a complex process which involves the division and proliferation of oogonia, and the development and maturation of oocytes [42]. During the process of oocyte maturation, many nutrients accumulate and store in oocytes, of which vitellin is the most important nutrient [43]. In most mollusks, Vitellogenin (Vg) is the major precursor of vitellin and is predominantly synthesized in the ovary and/or observed in the hepatopancreas [38]. In this study, both sequencing results and qRT-PCR analyses indicated that the relative expression of Vgs (Vg1 and Vg2) decreased in the ovaries of injected group (Figure 8 and Figure 9), which suggested that silencing of *Sj*ILPR down-regulated the transcription and translation of Vg in the ovary. After synthesis, Vg was eventually enzymatically cleaved to form vitellin subunits in the oocyte [44,45]. Cathepsin L1-like (CtsL1-like), a cysteine protease located in lysosomes, plays an important role in the hydrolysis of Vg, and processing of Vg into yolk proteins in the oocytes of oviparous species [46,47]. During ovarian development, the increase in CtsL1-like expression level correlated with the vitellogenesis process [48]. In our study, the expression of CtsL1-like was declined in the injected group, suggesting that *Sj*ILPR can promote the processing and deposition of yolk proteins in developing oocytes via affecting the expression of CtsL1-like. Besides Vg and CtsL1-like, the positive effect of Follistatin (FS) on ovarian development is well established in mollusks [37]. FS, a monomeric glycoprotein rich in cysteine, which is expressed at high levels in the reproductive tissues such as the ovary, plays role in oocyte cell maturation in zebrafish [49]. In our study, the relative expression of FS significantly decreased in the ovary after the silencing of *Sj*ILPR, indicating that *Sj*ILPR can promote ovarian development by regulating FS expression.

## 4. Materials and Methods

### 4.1. Animals

Adult healthy *S. japonica* were collected from the aquaculture station of Marine Fisheries Research Institute of Zhejiang in Xishan island (Zhoushan, Zhejiang, China). Ovarian development was classified into four stages (Stage I: oogonium production period, Stage II: protoplasmic growth period, Stage III: interstitial growth period, Stage IV: trophoplasmic growth period) according to the criteria of Lü et al. (2016b) [2]. Before tissue sample collection, animals were placed on ice for 30 min. All collected tissues were stored at −80 °C for RNA extraction.

### 4.2. Cloning and Sequence Analysis of SjILPR

Total RNA extraction was conducted using the Trizol reagent (Invitrogen, Carlsbad, CA, USA) following the manufacturer’s instructions, and qualified using a NanoDrop 2000 spectrophotometer (Thermo Fisher Scientific, Waltham, MA, USA). First-strand cDNA was synthesized using M-MLV reverse transcriptase (RNase H^−^) (TaKaRa Bio Inc., Japan). Cloning of the *Sj*ILPR partial fragment was conducted using the procedures as described in our previous study [4]. The gene-specific primers were designed based on de novo transcriptome sequencing [1] and are shown in Supplementary Table S1. The cDNA sequence of *Sj*ILPR was extended using 3′- and 5′-rapid amplification of cDNA ends (RACE). PCR products were separated by electrophoresis on 1.5% agarose gel, and then sequenced.

Once obtained, the complete *Sj*ILPR sequence was checked in a BLAST search to reveal homologies between our deduced sequence and homologs present in other species. The open reading frame (ORF) of *Sj*ILPR cDNA was predicted by the ORF Finder (https://www.ncbi.nlm.nih.gov/orffinder accessed on 20 July 2019) and then translated into amino acids sequence. The deduced protein sequence was analyzed using SMART (Simple Modular Architecture Research Tool) (http://smart.embl-heidelberg.de/ accessed on 25 July 2022) to predict conserved domains [50]. The three-dimensional (3D) structure of *Sj*ILPR was predicted using I-TASSER software [51] and edited using PyMOL viewer (Version 2.5).

### 4.3. Phylogenetic Analysis of SjILPR

The amino acid sequences of IRs and ILPRs were retrieved from the NCBI database. These sequences were aligned using ClustalW2 software (http://www.ebi.ac.uk/Tools/msa/clustalw2/ accessed on 26 July 2019), and a phylogenetic tree was constructed using the Neighbor-joining (NJ) method (1000 bootstrap replicates) in MEGA7.0 [52].

### 4.4. Subcellular Localization of SjILPR

To investigate the subcellular localization of *SjILPR*, HEK293 T cells were used as the in vitro model. Cell culture and transient transfection were generally according to Pang et al. (2019) [4]. HEK293 T cells were grown in Dulbecco’s modified Eagle’s medium (DMEM) supplemented with 10% FBS, 2% P/S under 37 °C, 5% CO_2_ in an incubator.

The recombinant plasmids were constructed as described in Lü et al. (2019) [3]. Co-transfection of the *SjILPR* and pEGFP-N1plasmid was carried out with lipofectamine 2000 (Invitrogen), according to manufacturer’s protocol. Four hours after transfection, cells were fixed with 4% formaldehyde at room temperature for 20 min. After washing twice with PBS, cytomembrane was stained with Dil for 20 min. Nuclei were then stained with DAPI for 10min. Subcellular localization of *SjILPR* was visualized using a digital confocal microscope (Leica TCSSP5, Heidelberg, Germany).

### 4.5. Expression of SjILPR in Different Tissue

The expression profile of *Sj*ILPR in different tissue was determined by real-time PCR (RT-qPCR). Eight tissues including the brain, liver, pancreas, muscle, heart, ovary, gill, and intestine were dissected from seven adult cuttlefish. Total RNA extraction and cDNA synthesis were performed as described above. Expression levels of *SjILPR* in different tissues were evaluated using TB Green™ PremixEx Taq™ II (TaKaRa Bio Inc., Japan). Gene-specific primers and internal reference gene (*β-actin*) were shown in Supplementary Table S1. The PCR reaction volume of 10 μL contained 0.4 μL cDNA template, 0.4 μL of each primer (10 μM), 5 μL TB Green™ Premix Ex Taq™ II, 0.2 μL ROX Reference Dye II, and 3.6 μL ultrapure water. The PCR reaction was initiated via denaturation at 94 °C for 10 min, followed by 40 cycles of 15 s at 95 °C, 45 s at 60 °C, and 2 min at 72 °C. All samples were analyzed in triplicate. The relative expression levels were calculated by the 2^−ΔΔCT^ method [53].

### 4.6. Expression of SjILPR during the Different Ovarian Development Stages of Female S. japonica

To evaluate the expression profile of *Sj*ILPR during the reproductive cycle of female *S. japonica*, tissues of the ovary, pancreas, and liver from four stages were collected and stored at −80 °C. The expression level of *Sj*ILPR was detected using qRT-PCR as described above.

### 4.7. RNA Interference (RNAi) In Vivo

Based on the nucleotide sequence of *Sj*ILPR obtained in the present study, two *SjILPR* specific-siRNAs (siRNA1 or siRNA2), and one scrambled siRNA were designed and synthesized by Sangon Biotech Co., Ltd., Shanghai, China (Supplementary Table S1).

For RNAi experiments, 32 cuttlefish were randomly divided into 4 groups (two control groups and two *Sj*ILPR -silenced groups). RNAi was carried out by injection of *Sj*ILPR siRNAs (100 μL, 2 μmol/mL) into individuals following the procedure described by Gore et al. (2005) [54]. Animals in control groups were injected with 100 μL scramble siRNA or normal saline (NS). To verify the effectiveness of RNAi, ovary tissues were dissected from four groups 48 h after injection and subjected to qRT-PCR analysis to determine the expression level of *Sj*ILPR. Therefore, the animals injected with siRNA with a high interference effect were used as the treatment group. Then ovarian tissues were collected from the control and treatment groups and subjected to transcriptome analysis. The sequencing, transcriptomic assembly, and gene annotation were performed by Novogene Bioinformatics Technology Company (Tianjin, China). Differentially expressed genes (DEG) of two group analysis was calculated with DESeqR package (1.10.1). The screening criteria used for target DEGs is as follows: [log2 (foldchange)] > 1 & q value < 0.005]. Simultaneously the statistical enrichment of DEGs in KEGG pathways was tested using KOBAS software [55].

Four down-regulated genes related to ovary development (Vitellogenin 1, Vitellogenin 2, Cathepsin L1-like, and Follistatin) were chosen for experimental validation by qRT-PCR. The primers for these genes are shown in Supplementary Table S1. qRT-PCR reactions were performed as described above.

### 4.8. Statistical Analysis

All quantitative data were shown as mean ± standard deviations (S.D.). Statistical differences were analyzed using one-way analysis of variance (ANOVA), followed by Duncan’s multiple range tests in SPSS 14.0. The statistical significance was set *p* < 0.05, as indicated by values with different small letters or asterisks.

## 5. Conclusions

In summary, we identified and characterized the ILPR gene in *S. japonica*, and sequence feature and phylogenetic analyses confirmed its identity and evolutionary status. Expression profiles of *Sj*ILPR among tissues and different ovarian development stages suggested its critical role in reproduction regulation. Comparative transcriptome analysis revealed that some ovarian development-related genes were significantly down-regulated after the silencing of *Sj*ILPR, further indicating its biological functions in ovarian development by influencing some pathways related to gonadal development. This work could provide valuable information to further investigate the molecular mechanism of the reproduction in mollusks.

## Figures and Tables

**Table 1:** Summary of sequencing reads after filtering.

Sample	Raw_Reads	Clean_Reads	Clean_Bases (Gb)	Q20 (%)	Q30 (%)	GC_pct (%)	Mapped_Reads
siRNA2-injected	71733238	70292362	10.54	96.77	91.78	38.48	55027712
Control	68396864	67258492	10.09	96.85	91.92	38.17	51430472

## Supplementary Materials

The following supporting information can be downloaded at: https://www.mdpi.com/article/10.3390/ijms232112903/s1.
