# Supplementary material for: Insulin-like Peptide Receptor (ILPR) in the Cuttlefish Sepiella japonica: Characterization, Expression, and Regulation of Reproduction
Source: Int J Mol Sci. 2022 Oct 26;23(21):12903. doi: 10.3390/ijms232112903 (PMC9654127; doi:10.3390/ijms232112903)
Supplement: Supplementary file 1 [file ijms-23-12903-s001.zip › ijms-1927838-supplementary.pdf]

Supplementary Material

1 Supplementary Tables

Table S1: Nucleotide sequences of primers and dsRNAs used

	Sequence (5'-3')	Usage
ILPR-F	ACAACAAGAGCCATTAAGC	cDNA fragment cloning
ILPR-R	GCAAGTTATGTCCGAGTATT	
ILPR 3'-F	TGCTTACTTGTTGGTCATAC	3'-RACE
ILPR 3'-R	CGCAATCAACAAGCAGAG	
ILPR 5'-F	GCCATTAGAAGCACAGACA	5'-RACE
ILPR 5'-R	TCTTACAGACGGCACCAT	
RT-ILPR-F	GGTGGAGATCACTGACTATC	Real-time PCR
RT-ILPR-R	CTCCACGCTGAATAACTGT	
RT-Vg1-F	CACCTGCGACTGAACCTAAA	qPCR for *Vg*1
RT-Vg1-R	CAAGACGCTCAAGCAACATG	
RT-Vg2-F	GAGTCAGGCTTGCTATGG	qPCR for *Vg*2
RT-Vg2-R	GTGGATTCATTACGGTCTAAG	
RT-CtsL1-like-F	GTATCTACTTACATACGCTGAC	qPCR for CtsL1-like
RT-CtsL1-like-R	AGTCCGAATAATGAGATGCT	
RT- FS-F	CTTGTCGTATGCGTGGAA	qPCR for FS
RT- FS-R	ATATAGGCGATACTGACTGAC	
RT-β-actin F	GCCAGTTGCTCGTTACAG	qPCR for β-actin
RT-β-actin R	GCCAACAATAGATGGGAAT	
negative control- sense	UUCUUCGAACGUGUCACGUTT	RNA interference
negative control- antisense	ACGUGACACGUUCGGAGAATT	
SjILPR siRNA1- sense	GCAAGUUAUGUCCGAGUAUTT	RNA interference
SjILPR siRNA1- antisense	AUACUCGGACAUAACUUGCTT	
SjILPR siRNA2- sense	GCUGGCUACAUUGAAGAUATT	RNA interference
SjILPR siRNA2- antisense	UAUCUUCAAUGUAGCCAGCTT	

Table S2. Accession numbers to the sequences in Multiple sequence alignment

Gene	Species	Accession numbers
SjILPR	*Sepiella japonica*	MK611806
ObILPR	*Octopus bimaculoides*	XP014780880.1
LsMIPR	*Lymnaea stagnalis*	CAA59353.1
BgIR	*Biomphalaria glabrata*	AAF31166.1
PfIRR	*Pinctada fucata*	AGA94627.1
AaIR	*Aedes aegypti*	AAB17094.1
BlILPR	*Branchiostoma lanceolatum*	AAB50848.1
HsINS	*Homo sapiens*	AAA59174.1

Table S3. List of accession numbers and identity of IRs from other species with *SjILPR*

Species	Accession numbers	Identities (%)
Sepiella japonica	MK611806	100
Octopus bimaculoides	XP014780880.1	67.3
Lymnaea stagnalis	CAA59353.1	39.2
Biomphalaria glabrata	AAF31166.1	36.6
Pinctada fucata	AGA94627.1	46.7
Aedes aegypti	AAB17094.1	35.6
Branchiostoma lanceolatum	AAB50848.1	41.6
Homo sapiens	AAA59174.1	40.7

Table S4. Summary of functional annotation of unigenes.

Database	Number.of.Unigenes	Percentage (%)
Annotated in NR	25602	24.82
Annotated in NT	8201	7.95
Annotated in KO	12276	11.9
Annotated in SwissProt	17718	17.18
Annotated in PFAM	28190	27.33
Annotated in GO	28190	27.33
Annotated in KOG	6637	6.43
Annotated in all Databases	2013	1.95
Annotated in at least one Database	37171	36.04
Total Unigenes	103117	100

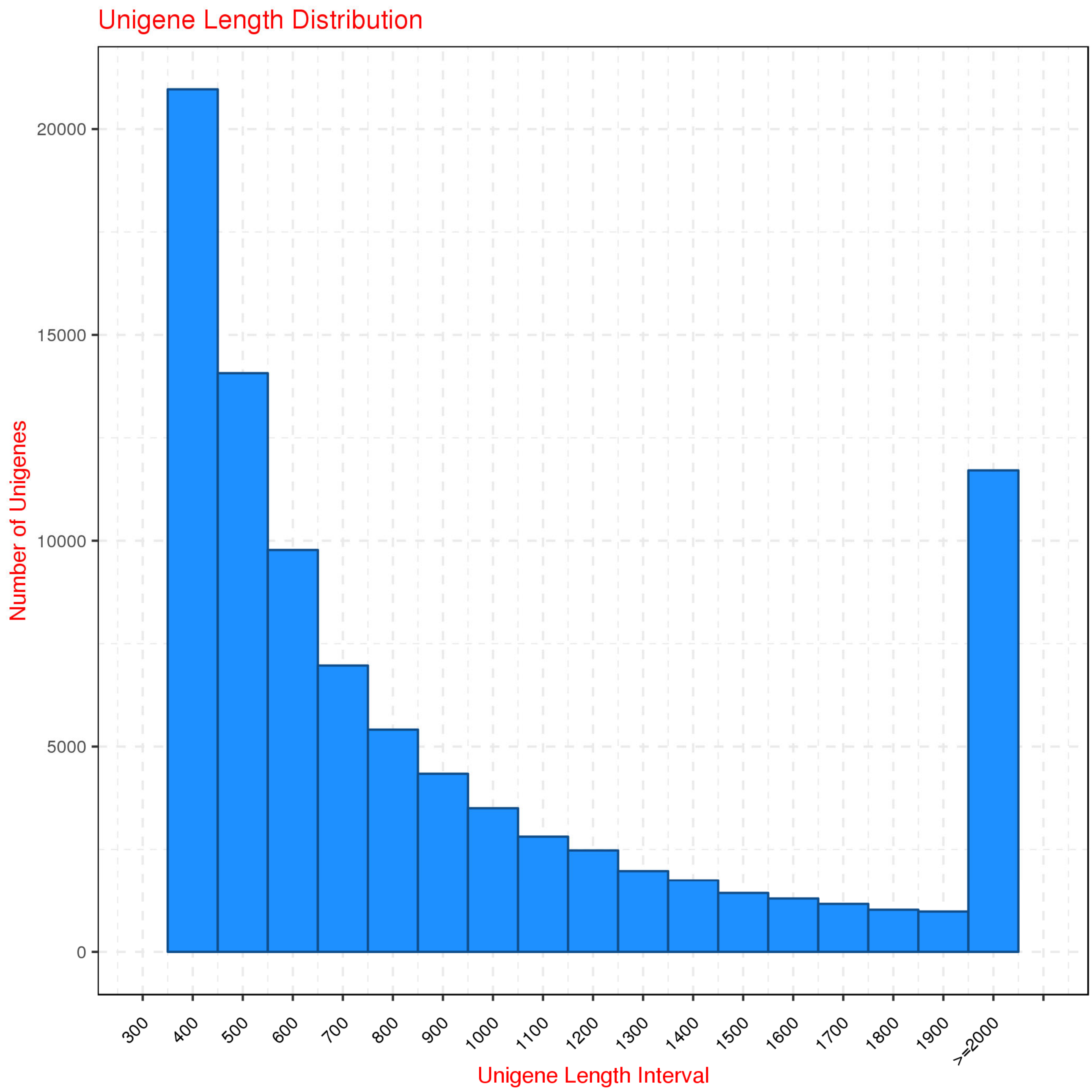

Figure S1. Length distribution of all-unigenes.
